# The Gut Microbiome in Schizophrenia and the Potential Benefits of Prebiotic and Probiotic Treatment

**DOI:** 10.3390/nu13041152

**Published:** 2021-03-31

**Authors:** Jonathan C. W. Liu, Ilona Gorbovskaya, Margaret K. Hahn, Daniel J. Müller

**Affiliations:** 1 Centre for Addiction and Mental Health, Campbell Family Mental Health Research Institute, Toronto, ON M5T 1R8, Canada; jonathan.liu@camh.ca (J.C.W.L.); ilona.gorbovskaya@camh.ca (I.G.); margaret.hahn@camh.ca (M.K.H.); 2 Department of Pharmacology and Toxicology, University of Toronto, Toronto, ON M5S 1A8, Canada; 3 Institute of Medical Sciences, University of Toronto, Toronto, ON M5S 1A8, Canada; 4 Department of Psychiatry, University of Toronto, Toronto, ON M5T 1R8, Canada; 5 Banting and Best Diabetes Centre, University of Toronto, ON M5G 2C4, Canada

**Keywords:** gut microbiome, gut–brain axis, schizophrenia, antipsychotics, prebiotics, probiotics, psychobiotics

## Abstract

The gut microbiome (GMB) plays an important role in developmental processes and has been implicated in the etiology of psychiatric disorders. However, the relationship between GMB and schizophrenia remains unclear. In this article, we review the existing evidence surrounding the gut microbiome in schizophrenia and the potential for antipsychotics to cause adverse metabolic events by altering the gut microbiome. We also evaluate the current evidence for the clinical use of probiotic and prebiotic treatment in schizophrenia. The current data on microbiome alteration in schizophrenia remain conflicting. Longitudinal and larger studies will help elucidate the confounding effect on the microbiome. Current studies help lay the groundwork for further investigations into the role of the GMB in the development, presentation, progression and potential treatment of schizophrenia.

## 1. Introduction

A relationship by which the gut microbiota (GMB) may interact with the central nervous system (CNS) through the gut–brain axis has long been construed. The gut–brain axis is a complex bilateral communication network that ensures proper maintenance of the gastrointestinal (GI) homeostasis. The fundamental mechanisms of the gut–brain axis communications involve neuro-immunoendocrine mediators through the CNS, the autonomic nervous system, the enteric nervous system and the hypothalamic pituitary adrenal axis [1]. In recent decades, studies have reported causal effects of the GMB on human behaviour and on the structure and function of the brain along with elucidating the underlying molecular mechanisms [2,3,4].

Current evidence indicates that communication from the GMB to the brain primarily occurs through neuroimmune and neuroendocrine mechanisms, often involving the vagus nerve [5]. This bottom-up communication is mediated by several microbial-derived molecules, with the best-elucidated examples including short-chain fatty acids, secondary bile acids and tryptophan metabolites [6,7,8]. While some of these microbial-derived molecules interact directly with enteroendocrine cells, enterochromaffin cells and the mucosal immune system, others may penetrate the intestinal barrier to enter systemic circulation. Although some of the molecules are able to cross the blood–brain barrier, it remains unclear whether they interact with brain sites directly or only through neural signaling via spinal and/or vagal afferents [5]. Additionally, the GMB can independently produce or contribute to the production of several neuroactive molecules such as serotonin (5HT), norepinephrine, dopamine and γ-aminobutyric acid [9,10,11]. However, it is unknown if they reach significant concentrations or relevant receptors to elicit a response. Through evolution, the human immune system has maintained a symbiotic relationship with the microbiota. The GMB was shown to play an important role in regulating various immune functions in mice such as autoimmunity, defense and inflammation. The GMB has been implicated in the development and function of the CNS immune cells, specifically microglia [12]. Relative to controls, GF mice have diminished microglia maturation leading to higher risk of pathogen exposure. Adult specific pathogen-free mice treated with antibiotics led to microglia re-entering immature status which can be rescued through recolonization with a complex microbiota. Treatment with antibiotics reverts microglia back to an immature state, suggesting continuous signaling of the GMB is needed for microglial function [12]. Disruption of the dynamic interaction between the two can result in profound effects on human health.

## 2. The Gut Microbiome in Behavior and Psychiatric Diseases

Several experimental approaches in animal models have been used to study the influence of the GMB on the gut–brain axis, including fecal microbial transplantation [13,14], colonization with human [14,15] or synthetic [16] microbiota, manipulation of the GMB with antibiotics [13,17,18] and probiotics [19,20,21] and germ-free (GF) animal models [13,22,23,24]. It was demonstrated that the absence of normal GMB in GF mice has significant effects on behaviour such as abnormal memory, sociability, anxiety-like behaviours and motor performance [23]. These behavioural abnormalities could be rescued by colonization at the neonatal stage. In addition to anxiety-like behaviours [19], the GMB is also implicated in relation to stress-responsiveness [19,22] and depression-like behaviours [25,26].

### 2.1. Schizophrenia

Schizophrenia (SCZ) is a psychiatric disorder with poorly defined molecular mechanisms with a prevalence of approximately 0.5% to 1% of the general population worldwide. In males, the age of onset ranges from 21 to 25 years, whereas in females, it ranges from 25 to 30 years [27]. Patients diagnosed with SCZ frequently experience a poor long-term prognosis, characterized by psychotic symptoms, poor social functioning and poor quality of life [28,29]. However, early treatment, regular monitoring, psychological interventions and social support can alleviate symptoms and lead to partial or full remission. The etiology of SCZ has yet to be fully elucidated; however, it is commonly accepted that many phenotypes of the disorder have a genetic or environmental influence [28]. The symptoms of SCZ are categorized as positive (presence of psychotic symptoms), negative (e.g., lack of motivation, social withdrawal) and cognitive (e.g., impaired abstract thinking). The Positive and Negative Syndrome Scale (PANSS) is a clinical scale used to measure symptom severity of patients with SCZ and AP treatment efficacy. Negative and cognitive symptoms are primarily responsible for the disabling features of SCZ, as they are significantly less responsive to antipsychotic medication treatment than positive symptoms [27,28]. Notably, some GI disturbances such as irritable bowel syndrome, inflammable bowel disease and celiac disease are prevalent comorbidities in SCZ [30]. Emerging preclinical and clinical studies indicate potential association between SCZ and the GMB. SCZ was proposed to be associated with chronic GI inflammation, metabolic dysfunction and oxidative stress [31].

Given the evidence implicating the GMB in behaviour and psychiatric diseases, it is possible that the GMB may play a role in SCZ through the gut–brain axis [32]. Studies have reported behaviours associated with SCZ which can be influenced by the GMB in animal studies such as social behaviour, mood and cognition. However, our literature search indicates that clinical studies investigating the relationship between SCZ and the GMB are limited. To date, twelve studies have been published investigating microbiome differences between individuals with SCZ and healthy controls (Table 1). We assessed human studies that evaluated the GMB of individuals with SCZ. We selected the MeSH terms: ‘“Microbiome” OR “Microbiota”’, AND ‘“Schizophrenia” OR “Antipsychotics”’ with the following filters: language: English; publication date: 2018—January 2021. The search was performed through PubMed. All articles were screened based on the title and abstract. Additional articles were screened and searched on the reference lists of the selected articles (Supplementary Figure S1).

### 2.2. Acute Schizophrenia

He et al. (2018) investigated the GMB in relation to the prodromal stage of SCZ and included ultra-high-risk subjects for SCZ, high-risk subjects and healthy controls [33]. Alpha and beta diversity analyses are used to measure the differences in GMB. Alpha diversity is a measure of the within-sample diversity typically expressed as the number (i.e., richness) or distribution (i.e., evenness) of bacteria in the sample. Whereas beta diversity is the measure of the between-sample microbial composition differences between two populations. He et al. (2018) reported no significant differences in alpha diversity between the groups. However, increased levels of the orders *Clostridiales, Lactobacillales and Bacteroidales* were observed in ultra-high-risk subjects for SCZ when compared to high-risk and healthy control subjects.

A few studies have looked into the GMB in antipsychotic-naïve patients with first-episode SCZ. One smaller study reported no significant differences in alpha diversity between the groups were found. However, principle coordinate analysis of beta diversity resulted in distinct clusters between SCZ patients and healthy controls, indicating an altered GMB in SCZ patients [36]. These findings were supported by another study which also found no significant differences in alpha diversity between antipsychotic-naïve patients with first-episode SCZ and healthy controls [43]. However, both groups were similar in relation to beta diversity. Both studies identified altered levels at various taxa, although there is some discord between these results, most likely due to the low sample size (n = 13) in study by Zhang et al. (2020). The phylum *Proteobacteria* was found to be both increased and similar in abundance in SCZ patients for the two studies [36,43]. However, differences in relative abundance at various taxa reported in the study by Zhang el al. [36] was not seen in the other study by Ma et al. [43]. Another study by Zhu et al. (2020) examined the GMB of medication-free first-episode SCZ patients using shotgun sequencing. Patients with SCZ were observed to have a higher alpha diversity and more variable GMB than controls [38]. Interestingly, they observed serum tryptophan levels were negatively correlated with the abundances of 26 bacterial species enriched in patients with schizophrenia [38]. 

### 2.3. Chronic Schizophrenia

Five studies have examined the GMB composition in chronically antipsychotic- treated SCZ patients and healthy controls indicating considerable differences among study designs and reported results. Zheng et al. (2019), Ma et al. (2020) and Xu et al. (2020) reported a significant decrease in GMB alpha diversity in SCZ patients as compared to healthy controls. However, Shen et al. (2018) and Nguyen et al. (2019) reported no differences between their comparable samples. While there is a consensus that SCZ patients present with an altered GMB compared to healthy controls, the evidence regarding specific taxa involved is inconsistent. For example, out of five studies, two studies reported an increased abundance of the phylum *Proteobacteria* [40,43], one study reported a decreased abundance [41] and two studies reported no significant difference when compared to healthy controls [42,44]. A couple studies investigated the oropharyngeal microbiota in SCZ patients. One study saw a lower alpha diversity in SCZ patients while the other study reported no significant differences [34,35]. Both studies observed an altered microbiome and differences at various taxa between groups, yet the findings were inconsistent between the two studies. Due to the small sample sizes in some of the studies, certain elevation and/or reduction of abundances might not have reached statistical significance. The precise microbial levels that are associated with SCZ would benefit from larger studies to account for the various factors that could affect the microbiome.

## 3. Antipsychotics and the Potential Role of the Gut Microbiome

### 3.1. Antipsychotic-Induced Metabolic Side Effects

Psychiatric patients have a higher relative risk of metabolic syndrome, a combination of cardiovascular risk factors such as dyslipidemia, hypertension, stroke and obesity, than the general population [45]. Moreover, the prevalence of metabolic side effects, type 2 diabetes and antipsychotic-induced weight gain (AIWG), is increased with the use of antipsychotics, specifically with olanzapine and clozapine [45,46,47]. Although the exact mechanisms by which metabolic dysfunction occurs remain unclear, several specific mechanisms of AIWG are supported by research evidence. One mechanism proposed is the antagonism of serotonergic and histaminergic receptors, resulting in increased appetite [48]. Additionally, genetic factors such as associations with 5HTR2C gene promoter polymorphisms or MC4R gene variants have been found to be associated with AIWG [48,49,50].

Evidence also suggests that peptide hormones might mediate AIWG as studies have found correlations between antipsychotics, weight gain and expression of peptide hormones such as leptin and C-peptide [51,52]. Antipsychotics can disrupt mitochondria function, related enzyme activity and ATP levels through downregulation in genes encoding subunits of the electron transport chain [53]. Our groups initiated a pilot study examining the effects of antipsychotics in patients with early psychosis, as well as in patients starting clozapine and in patients being chronically treated with clozapine in order to better understand the potential causal roles of antipsychotic medications and induced metabolic abnormalities [54].

The GMB has been proposed as a potential target in relation to AIWG and other metabolic dysfunction due to its ability to regulate metabolism, homeostasis and energy balance [55,56,57,58]. Morgan et al. (2014) demonstrated that the presence of a gut microbiome was necessary and sufficient for olanzapine-induced weight gain in GF mice. They proposed olanzapine’s effect on AIWG to be through its in vitro antimicrobial activity against resident enteric bacteria [59]. Sex may play a role as females have been observed to be more susceptible to metabolic side effects such as weight gain than males in both animal and human models, with some studies reporting a decrease in body weight in male rats [47,57,59,60,61]. Evidence suggests a lipogenic effect when administering olanzapine in rodents; however, significant weight gain is only seen in female rats [60]. Furthermore, co-treatment of minocycline with olanzapine prevents AIWG in mice. However, coadministration of tetracycline, a closely related antibiotic showed no effects on AIWG, suggesting the mechanism behind minocycline is distinct from its antibiotic properties [62]. Olanzapine was also observed to alter the GMB profile in both male and female-treated rats, specifically increased levels of *Firmicutes* and decreased levels of *Bacteriodetes* [55]. Furthermore, treatment with prebiotics or antibiotics attenuated the olanzapine-induced metabolic dysfunction, including AIWG in female rats [56,63]. However, Kao et al. (2018) observed no change in GMB composition after olanzapine treatment, which may have been due to the short duration and variable dose of olanzapine treatment. The prebiotic mixture alone was reported to have some notable effects such as increasing *Bifidobacteria* spp. and reducing species within the *Firmicutes* (*Coprococcus*, *Oscillibacter*, *C. coccoides*, *Roseburia intestinalis cluster*, *Clostridium XVIII cluster*) and *Proteobacteria* (*Escherichia*/*Shigella* spp.) phyla [63]. Furthermore, olanzapine was observed to attenuate this effect of the prebiotic mixture, which suggests the antipsychotic does influence the GMB.

### 3.2. Effects of Antipsychotics on the Microbiome

Variety of antipsychotics used in various research studies may play a role in this inconsistency as different antipsychotics have been shown to have different effects on the GMB [56,57,61]. A study examining the effects of APs on the GMB in patients with bipolar disorder found a significant decrease in species diversity in females [64]. Recent in vitro work extensively examined over 1000 marketed drugs and found that 24% of the drugs with human targets inhibited the growth of at least one strain [65]. Among these drugs, APs were overrepresented with 26 out of 37 having antibacterial activity and displayed a similar pattern when targeting specific species. The authors hypothesized that direct bacterial inhibition may not only be a side effect of the APs, but also be part of their mechanism of action [65]. However, clozapine, risperidone and olanzapine were reported to lack any antibacterial activity.

When investigating the GMB in first-episode SCZ patients, chronic SCZ patients and healthy controls, Ma et al. (2020) found that chronically antipsychotic-treated SCZ patients had increased abundance in family *Enterococcaceae* and *Lactobacillaceae* and in genus *Enterococcus*, *Escherichia*, *Lactobacillus*, *Shigella*, *Streptococcus* and *Veillonella* relative to first-episode SCZ patients [43]. The same microbiota levels were similar or increased in abundance in first-episode SCZ patients compared to healthy controls, suggesting that antipsychotics may have negative effects on microbiota levels. In a similar fashion, relative abundances of family *Peptostreptococcaceae* and *Veillonellaceae* and genus *Fusobacterium* and *Megasphaera* were increased in chronic SCZ patients compared to first-episode SCZ patients but remained unchanged between first-episode SCZ patients and healthy controls, suggesting a normalizing effect of antipsychotics on these microbiota levels [43].

Bahr et al. (2015) reported that chronic treatment of risperidone in male children (n = 18) was associated with a lower a *Bacteroidetes*:*Firmicutes* ratio and an increase in BMI. Furthermore, a gradual decrease in the *Bacteroidetes*:*Firmicutes* ratio was seen over the course of risperidone treatment [57].

Yuan et al. (2018) looked at the effects of risperidone in drug-naive, normal weight, first-episode SCZ patients. After a 24-week treatment plan, there were significant increases in relative abundance of fecal *Bifidobacterium* spp. and *Escherichia coli* and significant decreases in the abundance of fecal *Clostridium coccoides* group and *Lactobacillus* spp. Increases in body weight, BMI, blood-glucose, triglycerides and C-reactive protein were observed as well. Antipsychotic-naive patients with first-episode SCZ were found to have reduced levels of *Bifidobacterium* spp., *Escherichia coli* and *Lactobacillus* spp. and increased levels of *Clostridium coccoides* group compared to matched healthy controls. However, levels of *Lactobacillus* spp. and *Bifidobacterium* spp. were elevated in first-episode psychosis patients compared to controls following antipsychotic treatment [37,66]. Weight gain was found to be correlated with GMB composition of first-episode SCZ patients treated with risperidone. Specifically, increase in the relative abundance of *Bifidobacterium* spp. strongly correlated with weight gain.

However, another study conducted by Pelka-Wysiecka et al. (2019) showed differing results. The study analyzed the effect of olanzapine on the GMB in 20 SCZ patients [66]. Patients underwent a 7-day washout of all psychiatric medication, received standard hospital diet and were administered olanzapine treatment. Stool samples were taken at baseline after the washout and after 6 weeks of treatment. Similar to previous studies, only women experienced a significant increase in BMI. No significant changes were observed in alpha diversity and GMB composition [39]. However, the absence of significant changes may be due to the short intervention period.

Zhu et al. (2020) looked at the effects of APs on the GMB by following up with 38 medication-free patients with SCZ at baseline and 3 months after treatment. 26 microbial species were identified to differ between medication-free SCZ patients and HCs at baseline [38]. However, 20 species remained altered after 3 months of AP treatment compared with controls. Throughout AP treatment, authors reported 28 differentially abundant bacterial species, five of which were included in the 26 operational taxonomic unit SCZ classifiers [38]. The authors suggest that the GMB is influenced by APs but is not completely restored from SCZ-associated alterations.

Current clinical studies provide little evidence on the relationship between the APs, GMB and metabolic changes. Given the inconsistent findings highlighted in this review, future studies should ideally address the following: larger patient samples, with standardized medications, use of homogeneous populations (e.g., age, sex, BMI, symptom severity), comparable study designs and accounting for geographical diversity.

## 4. Evidence for Psychobiotic Intervention in Schizophrenia

A GMB is essential for optimal function of the immune system and immune dysfunction has been implicated in SCZ. Consequently, alterations in the GMB may play a key role in the etiology and treatment response of SCZ through bacterial infections and contributes to a chronic inflammatory state. The studies by Shen et al. (2018) and Zhang et al. (2020) found reduced levels of the genera: *Roseburia* and *Faecalibacterium* [40,41]. Notably, both genera play an important role in maintaining the intestinal barrier through the production of butyrate [67,68]. Braniste et al. (2014) concluded that not only the presence of a GMB is essential for normal development of the blood–brain barrier, but studies also indicated that the GMB can also regulate its permeability [69]. Therefore, through such mechanisms, disturbances to the GMB can lead to CNS infection and inflammation.

Although many findings were inconsistent, a few taxonomic groups were repeatedly reported to be altered in SCZ patients (Table 2). Multiple studies reported increased species within the *Fusobacterium*, *Lactobacillus*, *Megasphaera* and *Prevotella* genera, most of which are gram-negative bacteria [33,34,38,40,41,42,43,44]. While gram-negative bacteria are common in normal gut flora, increased permeability of the gut wall may result in systemic circulation of enteric inflammatory molecules such as lipopolysaccharides (LPS). An in vitro study examined the effects of LPS on intestinal epithelial cells and found that acute administration of LPS resulted in altered and reduced distribution of tight junctions [70]. LPS has been shown to be an effective neurodevelopmental model of SCZ in rodents [71,72,73]. In addition, to LPS, SCZ patients have been shown to have increased inflammatory cytokines which may contribute to the change in gut permeability and development of a “leaky gut” [74].

Notably, SCZ patients were reported to have lower levels of the family *Lachnospiraceae*, which has been reported to be beneficial in health such as butyrate and other short-chain fatty acid production. As previously mentioned, the *Lachnospiraceae* family includes *Roseburia* which contribute to the integrity of the intestinal barrier [67,68].

The idea of using prebiotics and probiotics to treat psychiatric symptoms, known as psychobiotics has been proposed by various studies [75]. Probiotics have shown to improve anxiety, cognition, neural activity, stress and signaling in both preclinical and clinical studies [19,22,76,77,78,79]. Depressive symptoms are also considered common clinical features in patients with SCZ and has been suggested to be associated with poor clinical outcomes in SCZ [80,81]. One study found that a combination of prebiotics, fructo-oligosaccharides and galacto-oligosaccharides, had anxiolytic and antidepressant-like effects when administered in mice [82]. There have been several reports of an altered GMB in animal models and patients with major depressive disorder [26,82,83,84,85]. However, the composition change observed varies and currently there is no consensus. Further investigations are needed to determine if targeting the GMB with psychobiotics can help with depressive symptoms and improve the clinical outcomes when treating SCZ.

Another study observed that prebiotics can alleviate some metabolic side effects associated with antipsychotic use. Kao et al. (2018) demonstrated the use of the prebiotic, Bimuno^TM^ galacto-oligosaccharides, as an adjunct to olanzapine treatment to attenuate the metabolic and weight disturbances in female rats [63]. They also reported a potential pro-cognitive action through an augmenting effect of the N-methyl-D-aspartate receptor.

In a randomized control trial, 58 patients with SCZ or schizoaffective disorder completed a 14-week probiotic treatment trial alongside their usual antipsychotic treatment [86]. The probiotic mixture contained *Lactobacillus rhamnosus* and *Bifidobacterium animalis*. Probiotic supplementation led to a reduction of the acute phase reactant von Willebrand factor in SCZ patients, suggested to be the secondary effect of probiotic-induced improvement of intestinal epithelium integrity. Patients treated with probiotics also were less likely to develop severe bowel difficulties [87]. Bowel difficulty was reported to be positively correlated with seropositivity of *Candida albicans* [88]. Positive symptoms of SCZ were significantly improved in males who were seronegative for *Candida albicans* compared to those who were seropositive within the 13-week timepoint. However, no significant effects on the PANSS scores were seen for the full duration of the study.

In a more recent study, probiotic *Bifidobacterium breve* A-1 was given to SCZ patients for 4 weeks and improvements were seen in anxiety, depression and PANSS scores [89]. Elevated levels of various interleukins were also measured, including IL-22 and tumour necrosis factor-related activation induced cytokine (TRANCE). The authors suggested that the reported symptom improvements could be due to the critical roles IL-22 and TRANCE play in the function of the gut epithelial barrier.

Ghaderi et al. (2019) investigated probiotic supplement treatment in SCZ patients by administering a combination of vitamin D and probiotic mixture containing *Bifidobacterium bifidum*, *Lactobacillus acidophilus*, *Lactobacillus fermentum* and *Lactobacillus reuteri* for 12 weeks [90]. A significant decrease in metabolic abnormalities and circulating C-reactive protein were noted, indicating reduced inflammation, alongside improvements in general and total PANSS scores and plasma total antioxidant capacity. However, it is uncertain whether vitamin D, probiotic supplement, or a combination of both are responsible for the observed improvements.

## 5. Conclusions

There are several limitations regarding the current evidence for the GMB, SCZ and psychobiotic-based treatments. The low sample size in most studies is a contributor to the variability of results reported as it can reduce the ability to detect smaller effects, increasing the risk of false negatives. While most studies used 16S rRNA gene sequencing for taxonomic identification, different primers and regions (V3 and V4) were used which have been reported to produce varying results [91]. Consequently, the conclusions drawn from the results should be viewed with caution. The different antipsychotic treatments used between patients may have contributed to the reported discrepancies. Therefore, longitudinal studies would aid in elucidating the effects of antipsychotics on the GMB composition mentioned previously. The use of antipsychotic treatment, specifically many second-generation antipsychotics are particularly likely to cause metabolic abnormalities and has been suggested to influence microbiota levels. Lifestyle factors such as dietary habits, smoking and obesity can also contribute to the inconsistent observations and should be carefully controlled for in future studies. Despite the inconclusive findings, some authors suggest that GMB dysbiosis could lead to SCZ-like outcomes; however, this suggested causal relationship needs to be further investigated. The use of prebiotics and probiotics to treat symptoms of SCZ and its comorbidities is promising, albeit it is still in its early stages. Larger scale studies with psychobiotic treatment at various stages of SCZ could help conclude the role of GMB in SCZ. Despite these limitations, these studies lay the groundwork for further investigations into the role of the GMB in the development, presentation and progression of SCZ.

## Figures and Tables

**Table 1:** Summary of studies investigating the microbiome in patients with schizophrenia.

Publication	Study Design	Diversity Findings	Taxa Abundance Differences in Schizophrenia
He et al., 2018 [33]	Gut microbiome 81 high-risk schizophrenia patients, 19 ultra-high-risk schizophrenia patients, 69 healthy controls Recruited from the Second Xiangya Hospital, Central South University, Changsha, Hunan, China **Experimental Method**: 16S rRNA gene sequencing from stool samples collected at baseline	No significant differences between the groups	Observed increased relative abundance of various taxa in schizophrenia: **Orders**: *Bacteroidales, Clostridiales, Lactobacillales* **Genera**: *Lactobacillus, Prevotella* **Species**: *Lactobacillus ruminis*
Castro-Nallar et al., 2015 [34]	Oral microbiome 16 schizophrenia patients, 16 healthy controls Recruited from the Stanley Research Program at Sheppard Pratt Hospital Maryland, US **Experimental Method**: Illumina sequencing of DNA from throat swabs	Lower species richness and more homogenously distributed in schizophrenia patients	Observed increased relative abundance of various taxa in schizophrenia: **Phyla**: *Firmicutes* **Genera**: *Bifidobacterium*, *Lactobacillus* **Species**: *Bifidobacterium pseudocatenulatum, Candida dubliniensis, Catenibacterium mitsuokai, Eubacterium hallii, Lactobacillus gasseri, Lactobacillus salivarius*
Yolken et al., 2020 [35]	Oral microbiome 121 schizophrenia patients, 85 healthy controls Recruited from psychiatric programs affiliated with the Sheppard Pratt Health System and at other outpatient treatment sites in central Maryland, US **Experimental Method**: 16S rRNA gene sequencing of V3–V4 region from throat swabs	No significant differences in alpha diversity (microbial richness and diversity) were found Altered beta diversity was found in individuals with schizophrenia compared to healthy controls	Observed increased relative abundance of various taxa in schizophrenia: **Genera**: *Streptococcus* Observed decreased relative abundance of various taxa in schizophrenia: **Families**: *Weeksellaceae* **Genera**: *Prevotella* **Species**: *Neisseria subflava*
Zhang et al., 2020 [36]	Gut microbiome 10 antipsychotic-naïve patients with first-episode schizophrenia, 16 healthy controls Recruited from the Seventh People’s Hospital of Hangzhou in Hangzhou, Zhejiang **Experimental Method**: 16S rRNA gene sequencing from stool samples collected at baseline	No significant differences in alpha diversity (microbial richness and diversity) were found Principle coordinate analysis distinguished a cluster between the two groups, indicating a significant decrease in beta diversity in the schizophrenia group compared to the control group	Observed increased relative abundance of various taxa in schizophrenia: **Phyla**: *Proteobacteria* **Classes**: *Deltaproteobacteria, Saccharimonadia, Synergistia* **Orders**: *Actinomycetales, Desulfovibrionales, Saccharimonadales, Synergistales* **Families**: *Actinomycetaceae, Burkholderiaceae, Desulfovibrionaceae, Saccharimonadaceae, Synergistaceae* **Genera**: *Actinomyces, Anaerotruncus, Bilophila, Blautia, Christensenella, Cloacibacillus, Dorea, Eggerthella, Eisenbergiella, Flavonifractor, Holdemania, Hungatella, Oscillibacter, Parasutterella, Prevotella* Observed decreased relative abundance of various taxa in schizophrenia: **Families**: *Lachnospiraceae* **Genera**: *Agathobacter, Butyricicoccus, Coprococcus, Faecalibacterium, Fusicatenibacter, Ruminococcus*
Yuan et al., 2018 [37]	Gut microbiome 41 antipsychotic naïve patients with first-episode schizophrenia and 41 healthy controls Patients were started on risperidone treatment for 24 weeks (1–6 mg/day) Recruited from First Affiliated Hospital of Zhengzhou University **Experimental Method**: 16S rRNA gene sequencing from stool samples collected from baseline, weeks 6, 12 and 24	Not reported	Abundance of *Bifidobacterium* spp. and *Escherichia coli* increased with risperidone treatment Abundance of *Lactobaccillus* spp. and *Clostridium coccoides* decreased with risperidone treatment Observed increased relative abundance of various taxa in schizophrenia: **Species**: *Clostridium coccoides* Observed decreased relative abundance of various taxa in schizophrenia: **Genera**: *Bifidobacterium*., *Lactobacillus* **Species**: *Escherichia coli*
Zhu et al., 2020 [38]	Gut microbiome 90 antipsychotic-free patients with first-episode schizophrenia and 81 healthy controls Followed up with 38 patients after 3 months of treatment (27 risperidone and 11 other antipsychotics) Recruited from multiple clinical sites across the Shaanxi Province, China **Experimental Method**: Metagenomic shotgun sequencing from stool samples collected at baseline and 3 months after antipsychotic treatment	Higher alpha diversity (microbial richness and diversity) was observed at the genus level Individuals with schizophrenia presented with a more variable gut microbiome compared to healthy controls	Observed increased relative abundance of various **Species** in chronically antipsychotic-treated schizophrenia compared to: **HC***: Acidaminococcus fermentans, Acidaminococcus intestini, Akkermansia muciniphila, Alkaliphilus oremlandii, Bacillus amyloliquefaciens, Bacteroides plebeius, Bifidobacterium adolescentis, Bifidobacterium bifidum, Bifidobacterium dentium, Bifidobacterium longum, Enterococcus faecium, Eubacterium siraeum, Lactobacillus casei, Lactobacillus crispatus, Lactobacillus delbrueckii, Lactobacillus fermentum, Lactobacillus oris, Lactobacillus ruminis, Lactobacillus salivarius, Methanobrevibacter smithii, Pseudoflavonifractor capillosus, Stenotrophomonas maltophilia, Streptococcus anginosus, Streptococcus mutans, Veillonella atypica, Veillonella parvula* **FSCZ***: Actinomyces odontolyticus, Enterobacter aerogenes, Enterobacter asburiae, Enterobacter cancerogenus, Enterobacter cloacae, Escherichia coli, Lactococcus lactis, Victivallis vadensis,* Observed decreased relative abundance of various **Species** in chronically antipsychotic-treated schizophrenia compared to: **HC**: *Bacteroides intestinalis, Lactobacillus acidophilus, Lactococcus lactis* **FSCZ**: *Alkaliphilus oremlandii, Anaerostipes caccae, Bacteroides ovatus, Bifidobacterium angulatum, Bifidobacterium bifidum, Bifidobacterium longum, Clostridium bolteae, Dorea formicigenerans, Enterococcus faecium, Eubacterium hallii, Faecalibacterium prausnitzii, Lactobacillus ruminis, Streptococcus anginosus* Observed increased relative abundance of various **Genera** in first-episode schizophrenia compared to controls: *Acidaminococcus, Akkermansia, Anaerotruncus, Bifidobacterium, Citrobacter, Clavibacter, Comamonas, Coprobacillus, Cryptobacterium, Dialister, Enterococcus, Lactobacillus, Methanobrevibacter, Peptoniphilus, Pseudoflavonifractor, Veillonella* Observed decreased relative abundance of various **Genera** in first-episode schizophrenia compared to controls: *Butyrivibrio, Gemella*
Pełka-Wysiecka et al., 2019 [39]	Gut microbiome 20 schizophrenia patients Patients underwent a 7 day washout and were started on olanzapine for 2 weeks (5–20 mg/day). Recruited from Department of Psychiatry in Szczecin (Poland) **Experimental Method**: 16S rRNA gene sequencing of V4 region from stool samples collected after washout and week 2 of treatment.	No significant changes in alpha diversity after 2 weeks of olanzapine treatment	No significant changes in OTU abundancies between weeks 0 and 6. No changes in Firmicutes/Bacteroidetes ratio
Shen et al., 2018 [40]	Gut microbiome 64 schizophrenia patients on antipsychotic medication and 53 healthy controls Recruited from hospital or outpatient clinics within Huludao area in China **Experimental Method**: 16S rRNA gene sequencing of V3–V4 region from stool samples collected at baseline	No significant differences in alpha diversity (microbial richness and diversity) were found	Observed increased relative abundance of various taxa in schizophrenia: **Phyla**: *Proteobacteria* **Classes**: *Gammaproteobacteria* **Orders**: *Aeromonadales, Fusobacteriales* **Families**: *Enterobacteriaceae, Fusobacteriaceae, Lactobacillaceae, Prevotellaceae, Succinivibrionaceae, Veillonellaceae* **Genera**: *Acidaminococcus, Citrobacter, Clostridium, Collinsella, Desulfovibrio, Fusobacterium, Klebsiella, Lactobacillus, Megasphaera, Phascolarctobacterium, Prevotella, Succinivibrio* **Species**: *Bifidobacterium adolescentis, Bacteroides fragilis, Collinsella aerofaciens, Lactobacillus mucosae, Prevotella stercorea* Observed decreased relative abundance of various taxa in schizophrenia: **Phyla**: *Firmicutes* **Classes**: *Clostridia* **Orders**: *Clostridiales* **Families**: *Alkaligenaceae, Lachnospiraceae* **Genera**: *Blautia, Coprococcus, Roseburia, Streptococcus* **Species**: *Bacteroides eggerthii, Blautia producta, Collinsella plebeius, Roseburia faecis*
Nguyen et al., 2019 [41]	Gut microbiome 25 patients with chronic schizophrenia or schizoaffective disorder (most patients were being treated with antipsychotic medication), 25 demographically matched non-psychiatric controls Recruited as outpatients in San Diego **Experimental Method**: 16S rRNA gene sequencing of V4 region from stool samples collected at baseline	No significant differences in alpha diversity (microbial richness and diversity) were found	Observed increased relative abundance of various taxa in schizophrenia: **Genera**: *Anaerococcus, Blautia, Megasphaera,, Ruminococcus* Observed decreased relative abundance of various taxa in schizophrenia: **Phyla**: *Proteobacteria* **Genera**: *Clostridium, Haemophilus, Oscillospira, Sutterella* **Species**: *Haemophilus parainfluenzae*
Zheng et al., 2019 [42]	Gut microbiome 63 schizophrenia patients (most patients were being treated with antipsychotic medication), 69 healthy controls Recruited from the First Affiliated Hospital of Chongqing Medical University **Experimental Method**: 16S rRNA gene sequencing of V3–V4 region from stool samples collected at baseline	Lower alpha diversity (species richness and diversity) was observed in schizophrenia patients	Observed increased relative abundance of various taxa in schizophrenia: **Families**: *Bacteroidaceae, Coriobacteriaceae*, *Prevotellaceae, Veillonellaceae* **Genera**: *Akkermansia, Fusobacterium, Megasphaera, Prevotella* Observed decreased relative abundance of various taxa in schizophrenia: **Families**: *Acidaminococcaceae, Enterobacteriaceae, Lachnospiraceae, Rikenellaceae, Ruminococcaceae* **Genera**: *Blautia, Citrobacter, Coprococcus, Lachnoclostridium* **Species**: *Bacteroides eggerthii, Bacteroides massiliensis, Collinsella stercoris, Haemophilus parainfluenzae*
Ma et al., 2020 [43]	Gut microbiome 40 antipsychotic-naïve patients with first-episode schizophrenia (FSCZ), 85 chronically antipsychotic-treated schizophrenia patients, 69 healthy controls (HC) Recruited from the Second Xiangya Hospital of Central South University **Experimental Method**: 16S rRNA gene sequencing of V4 region from stool samples collected at baseline	Lower alpha diversity (species richness and diversity) was observed in chronically antipsychotic-treated schizophrenia patients compared to antipsychotic-naïve patients with first-episode schizophrenia and healthy controls	Observed increased relative abundance of various taxa in chronically antipsychotic-treated schizophrenia compared to: **Phyla** **HC**: *Proteobacteria* **Families** **HC**: *Christensenellaceae, Enterobacteriaceae, Enterococcaceae, Lactobacillaceae* **FSCZ**: *Enterococcaceae, Lactobacillaceae, Peptostreptococcaceae. Streptococcaceae, Veillonellaceae* **Genera** **HC**: *Escherichia, Bulleidia, Coprobacillus, Enterococcus, Lactobacillus, Shigella, Streptococcus, Trabulsiella, Veillonella* **FSCZ**: *Citrobacter, Clostridium, Enterobacter, Enterococcus, Escherichia, Fusobacterium, Lachnobacterium, Megasphaera, Lactobacillus, Ruminococcus, Shigella, Streptococcus, Sutterella, Veillonella* Observed decreased relative abundance of various taxa in chronically antipsychotic-treated schizophrenia compared to: **Phyla** **HC**: *Cyanobacteria* **FSCZ**: *Lentisphaerae* **Families** **HC**: *Pasteurellaceae, Turicibacteraceae* **Genera** **HC**: *Bacteroides, Parabacteroides, Turicibacter* **FSCZ**: *Lachnobacterium*
Xu et al., 2020 [44]	Gut microbiome 44 schizophrenia patients, 44 healthy controls Recruited from Longgang Central Hospital of Shenzhen and Shenzhen Kangning Hospital in Shenzhen, China **Experimental Method**: 16S rRNA gene sequencing of V4 region from stool samples collected at baseline	Lower species richness was observed in schizophrenia patients NMDS analysis at the species level resulted in distinct clusters with few overlaps between schizophrenia patients and healthy controls. The microbial dysbiosis index was significantly increased in patients with schizophrenia compared to controls.	Observed increased relative abundance of various taxa in schizophrenia: **Phyla**: *Actinobacteria* **Classes**: *Deltaproteobacteria* **Orders**: *Actinomycetales, Sphingomonadales* **Families**: *Sphingomonadaceae* **Genera**: *Eggerthella, Megasphaera* **Species**: *Akkermansia muciniphila, Bifidobacterium adolescentis, Clostridium perfringens, Lactobacillus gasseri, Megasphaera elsdeniis* Observed decreased relative abundance of various taxa in schizophrenia: **Orders**: *Rhodocyclales* **Families**: *Alcaligenaceae, Enterococcaceae, Leuconostocaceae, Rhodocyclaceae, Rikenellaceae* **Genera**: *Enterococcus*

Experimental Methods and Taxonomic Groups are in bold.

**Table 2:** List of taxonomic groups that were significantly increased/decreased in multiple studies.

Taxa	Increased in Schizophrenia	Decreased in Schizophrenia
Phylum: *Firmicutes*	Castro-Nallar et al., 2015 (Oral) [34]	Shen et al., 2018 [40]
Phylum: *Proteobacteria*	Shen et al., 2018 [40] Ma et al., 2020 (vs HC only) [43] Zhang et al., 2020 (FSCZ) [36]	Nguyen et al., 2019 [41]
Class: *Deltaproteobacteria*	Xu et al., 2020 [44] Zhang et al., 2020 (FSCZ) [36]	
Order: *Actinomycetales*	Xu et al., 2020 [44] Zhang et al., 2020 (FSCZ) [36]	
Order: *Clostridiales*	He et al., 2018 (High-risk for SCZ) [33]	Shen et al., 2018 [40]
Family: *Enterobacteriaceae*	Shen et al., 2018 [40] Ma et al., 2020 (vs. HC only) [43]	Zheng et al., 2019 [42]
Family: *Enterococcaceae*	Ma et al., 2020 [43]	Xu et al., 2020 [44]
Family: *Lactobacillaceae*	Shen et al., 2018 [40] Ma et al., 2020 [43]	
Family: *Lachnospiraceae*		Shen et al., 2018 [40] Zheng et al., 2019 [42] Zhang et al., 2020 (FSCZ) [36]
Family: *Prevotellaceae*	Shen et al., 2018 [40] Zheng et al., 2019 [42]	
Family: *Rikenellaceae*	Zheng et al., 2019	Xu et al., 2020 [44]
Family: *Veillonellaceae*	Shen et al., 2018 [40] Zheng et al., 2019 [42] Ma et al., 2020 (vs. FSCZ only) [43]	
Genus: *Acidaminococcus*	Shen et al., 2018 [40] Zhu et al., 2020 (FSCZ) [38]	
Genus: *Akkermansia*	Zheng et al., 2019 [42] Zhu et al., 2020 (FSCZ) [38]	
Genus: *Anaerotruncus*	Zhang et al., 2020 (FSCZ) [36] Zhu et al., 2020 (FSCZ) [38]	
Genus: *Bifidobacterium*	Castro-Nallar et al., 2015 (Oral) [34] Zhu et al., 2020 (FSCZ) [38]	Yuan et al., 2018 (FSCZ) [37]
Genus: *Blautia*	Nguyen et al., 2019 [41] Zhang et al., 2020 (FSCZ) [36]	Shen et al., 2018 [40] Zheng et al., 2019 [42]
Genus: *Citrobacter*	Shen et al., 2018 [40] Ma et al., 2020 (vs. FSCZ only) [43] Zhu et al., 2020 (FSCZ) [38]	Zheng et al., 2019 [42]
Genus: *Clostridium*	Shen et al., 2018 [40] Ma et al., 2020 (vs. FSCZ only) [43]	
Genus: *Coprobacillus*	Ma et al., 2020 (vs. HC only) [43] Zhu et al., 2020 (FSCZ) [38]	
Genus: *Coprococcus*	Zhang et al., 2020 (FSCZ) [36]	Shen et al., 2018 [40] Zheng et al., 2019 [42]
Genus: *Eggerthella*	Xu et al., 2020 [44] Zhang et al., 2020 (FSCZ) [36]	
Genus: *Enterococcus*	Ma et al., 2020 [43] Zhu et al., 2020 (FSCZ) [38]	Xu et al., 2020 [44]
Genus: *Fusobacterium*	Shen et al., 2018 [40] Zheng et al., 2019 [42] Ma et al., 2020 (vs. FSCZ only) [43]	
Genus: *Lactobacillus*	Castro-Nallar et al., 2015 (Oral) [34] He et al., 2018 (High-risk for SCZ) [33] Shen et al., 2018 [40] Ma et al., 2020 [43] Zhu et al., 2020 (FSCZ) [38]	Yuan et al., 2018 (FSCZ) [37]
Genus: *Megasphaera*	Shen et al., 2018 [40] Nguyen et al., 2019 [41] Zheng et al., 2019 [42] Ma et al., 2020 (vs. FSCZ only) [43] Xu et al., 2020 [44]	
Genus: *Prevotella*	He et al., 2018 (High-risk for SCZ) [33] Shen et al., 2018 [40] Zheng et al., 2019 [42] Zhang et al., 2020 (FSCZ) [36]	Yolken et al., 2020 (Oral) [35]
Genus: *Ruminococcus*	Nguyen et al., 2019 [41] Ma et al., 2020 (vs. FSCZ only) [43]	Zhang et al., 2020 (FSCZ) [36]
Genus: *Streptococcus*	Ma et al., 2020 [43] Yolken et al., 2020 (Oral) [35]	Shen et al., 2018 [40]
Genus: *Veillonella*	Ma et al., 2020 [43] Zhu et al., 2020 (FSCZ) [38]	
Species: *Akkermansia muciniphila*	Xu et al., 2020 [44] Zhu et al., 2020 (vs. HC only) [38]	
Species: *Bacteroides eggerthii*		Shen et al., 2018 [40] Zheng et al., 2019 [42]
Species: *Bifidobacterium adolescentis*	Shen et al., 2018 [40] Xu et al., 2020 [44] Zhu et al., 2020 (vs. HC only) [38]	
Species: *Escherichia coli*	Zhu et al., 2020 (vs. FSCZ only) [38]	Yuan et al., 2018 (FSCZ) [37]
Species: *Eubacterium hallii*	Castro-Nallar et al., 2015 (Oral) [34]	Zhu et al., 2020 (vs FSCZ only) [38]
Species: *Lactobacillus gasseri*	Castro-Nallar et al., 2015 (Oral) [34] Xu et al., 2020 [44]	
Species: *Lactobacillus ruminis*	He et al., 2019 (High-risk for SCZ) [33] Zhu et al., 2020 (vs. HC only) [38]	Zhu et al., 2020 (vs FSCZ) [38]
Species: *Lactobacillus salivarius*	Castro-Nallar et al., 2015 (Oral) [34] Zhu et al., 2020 (vs. HC only) [38]	
Species: *Haemophilus parainfluenzae*		Nguyen et al., 2019 [41] Zheng et al., 2019 [42]

## Data Availability

No new data were created or analyzed in this study. Data sharing is not applicable to this article.

## Supplementary Materials

The following are available online at https://www.mdpi.com/article/10.3390/nu13041152/s1, Figure S1: Flowchart of study search and selection.
