# Supplementary figures and images for: The Gut Microbiome in Schizophrenia and the Potential Benefits of Prebiotic and Probiotic Treatment
Source: Nutrients. 2021 Mar 31;13(4):1152. doi: 10.3390/nu13041152 (PMC8065775; doi:10.3390/nu13041152)

Supplementary Figure S1: Flowchart of study search and selection

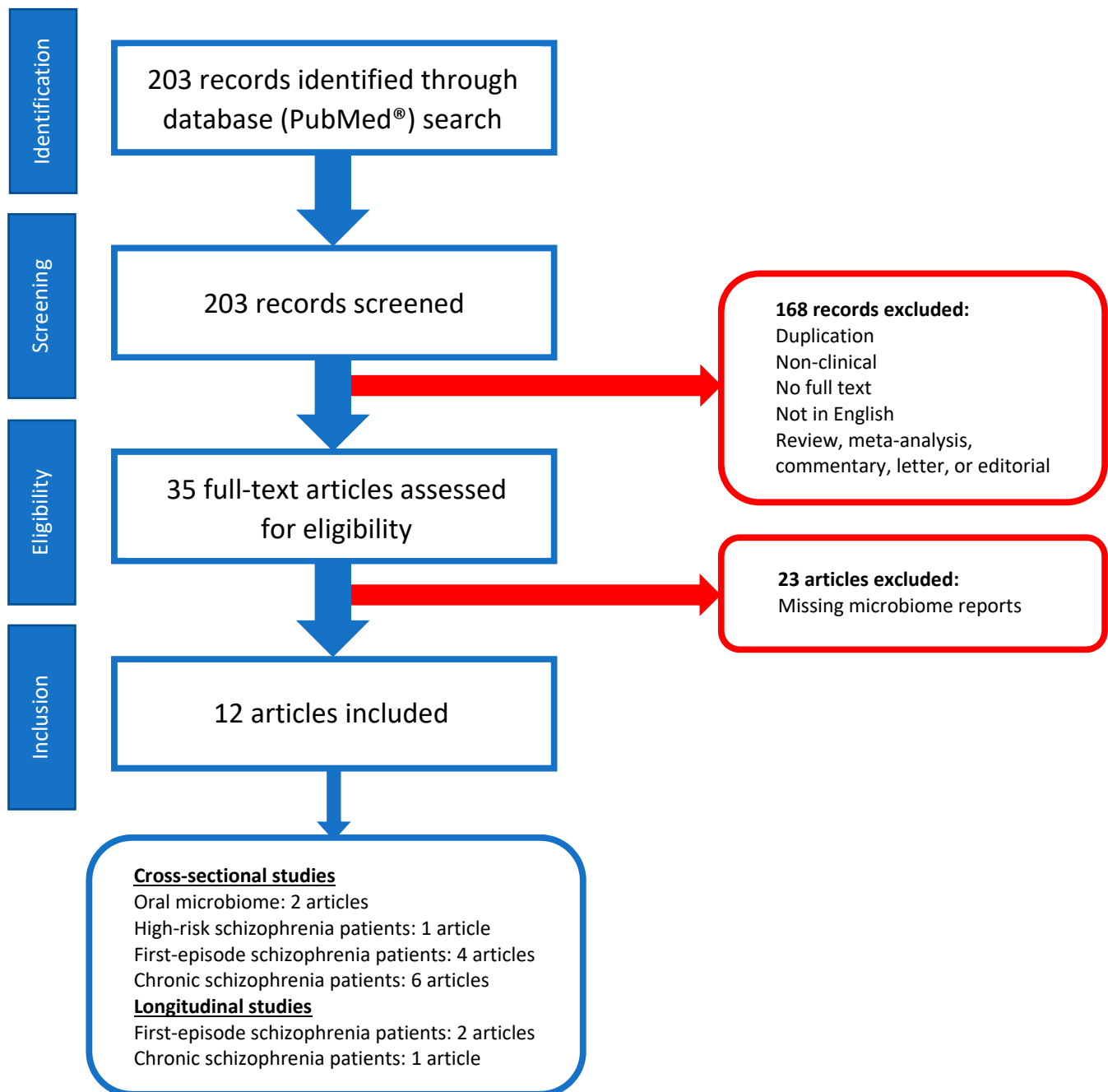

Supplement: Supplementary file 1 [file nutrients-13-01152-s001.pdf]
